# PDA modification and properties of α-AlH_3_

**DOI:** 10.1038/s41598-022-16424-8

**Published:** 2022-07-19

**Authors:** Mingna Qin, Bingjie Yao, Qiang Shi, Wang Tang, Shaoli Chen, Tao Guo, Wei Wang, Yan Zhang, Zhongxue Ge

**Affiliations:** 1 Xi’an Modern Chemistry Research Institute, Xi’an, 710065 Shaanxi People’s Republic of China; 2 Fujian Polytechnic Normal University, Fuzhou, 363000 Fujian People’s Republic of China

**Keywords:** Chemistry, Energy science and technology, Materials science

## Abstract

We present a novel surface coating to resolve the stability of α-AlH_3._ Inspired by the strong chemical adhesion of mussels, the polymerization of dopamine was first introduced to coat α-AlH_3_ through simple situ polymerization. The α-AlH_3_ was used as a substrate. In-depth characterizations confirmed the formation of polydopamine (PDA) on the α-AlH_3_ surface. The coated α-AlH_3_ sample was characterized by X-ray diffraction X-ray photoelectron spectrometry and Scanning Electron Microscope. The results show that a strong PDA film is formed on the surface of α-AlH_3_, and PDA@α-AlH_3_ retains its primary morphology. The crystal form of α-AlH_3_ does not change after coating with PDA. The XPS analysis results show that N1 s appears on the material after coating with PDA, indicating that polydopamine is formed on the surface of α-AlH_3_. The moisture absorption tests show that the moisture absorption rate of α-AlH_3_ is greatly reduced after being coated with PDA. The excellent intact ability of PDA prevents α-AlH_3_ from reacting with water in air. The thermal stability of α-AlH_3_ before and after coating was analyzed by DSC. This work demonstrates the successful applications of dopamine chemistry to α-AlH_3_, thereby providing a potential method for metastable materials.

## Introduction

AlH_3_ is a solid-state hydrogen storage, hydrogen provider and reducing agent. Combined with Ammonium dinitramide (ADN), it becomes a high energy material often used for aerospace and missile industry^1–4^. Theoretical studies have shown that the specific impulse value of AlH_3_ is higher in solid, liquid and solid–liquid propellants than Al^5–7^. AlH_3_ is a special compound. It has at least seven different crystalline structures depending on the synthesis conditions: α α, β γ δ ε and ζ. Among them, α-AlH_3_ is the best-studied crystalline^8–12^. However, α-AlH_3_ has a small enthalpy of formation and is in a metastable state. Hence, the problem related to stability remains unresolved^13–16^.

To date, various researchers in the worldwide have been tackling this problem in improving the stability of AlH_3_, including surface passivation, surface coating and doping with other substrates^17–24^. In spite of this, the appropriate coating material and the novel coating technique for coating α-AlH_3_ should be explored.

Dopamine is a biological neurotransmitter that widely exists in living organisms^25^. The use of dopamine solution through the oxidation-polymerization of monomers has provided a facile and versatile method for modifying the surfaces of solid materials, which has led to the development of bioinspired PDA for the successful modification of various substrates, including metals, metals with native oxide surfaces, ceramics, semiconductors, carbon materials, and synthetic polymers. PDA-mediated chemistry could provide a general method for the fabrication of numerous multifunctional substrates with specific properties^26–30^. Dopamine chemistry is a straightforward and versatile coating strategy that may open a door for the surface processing of α*-*AlH_3._ However, few works have reported the use of PDA coating on α-AlH_3._

Herein, we report a general and facile approach to the coating of α-AlH_3,_ which is bioinspired through the in situ polymerization of dopamine. To the best of our knowledge, this is the first report about the application of dopamine chemistry to α-AlH_3_. First, dopamine was added to the phosphate buffer saline (PBS). The as-prepared α-AlH_3_ then underwent dopamine polymerization through immersion in a freshly prepared dopamine solution, with stirring at 200 rpm for 4 h ^30^. A simple scatter of α-AlH_3_ in an aqueous dopamine solution can lead to the spontaneous deposition of PDA film. The stability of α-AlH_3_ is significantly improved through the PDA coating. The present work potentially provides a new method for the modification of metastable.

## Results and discussion

**Figure 1:**
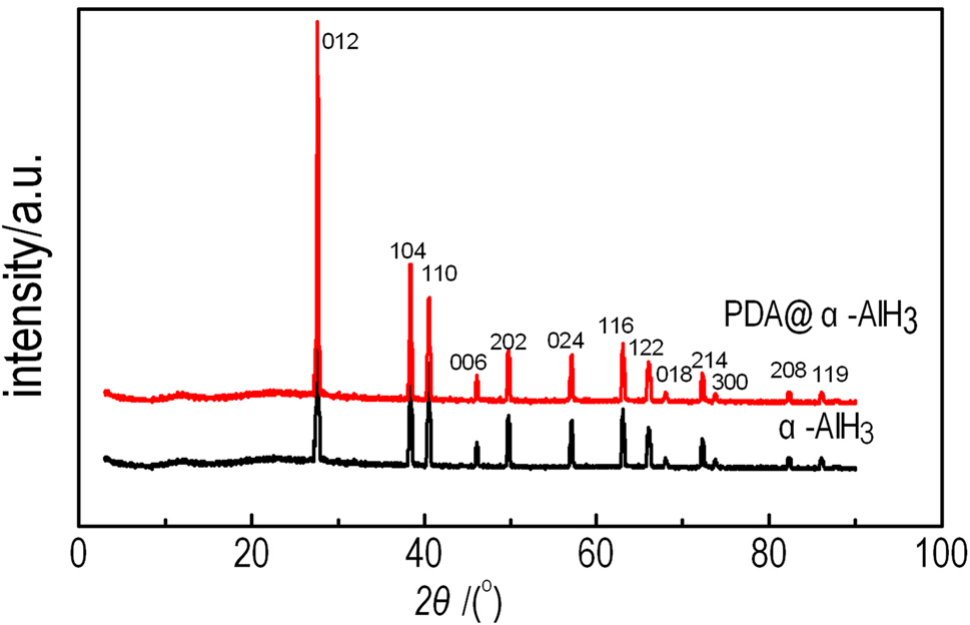
XRD patterns of PDA@α-AlH_3_ and α-AlH_3_.

**The** PDA@α-AlH_3_ composite and α-AlH_3_ were subjected to XRD analysis, and the results are shown in Fig. 1. Figure 1 shows that the characteristic peaks of the PDA was amorphous without the characteristic diffraction peak of PDA. composite material appear at 2*θ* = 27.84°, 38.58°, 40.72°, 46.1°, 49.96°, 57.26°, 63.26°, 66.26°, 68.14°, 72.48°, 73.84°, 82.52°, 86.16°, corresponding to the (012), (104), (006), (202), (024), (116), (122), (018), (214), (300), (208) and (119) planes of α-AlH_3_ (JCPDF 23-0761), respectively. The position of the diffraction peak of the PDA@α-AlH_3_ complex is basically the same as the characteristic diffraction peak of α-AlH_3_. It shows that the position of the characteristic diffraction peak remains unchanged before and after the modification, indicating that α-AlH_3_ has good crystallization performance and PDA was amorphous without the characteristic diffraction peak of PDA. Observed changes in the relative peak intensities are attributed to the decrease of particle crystallinity andthe increasedscattering power caused by the coated PDA^18^.

**Figure 2:**
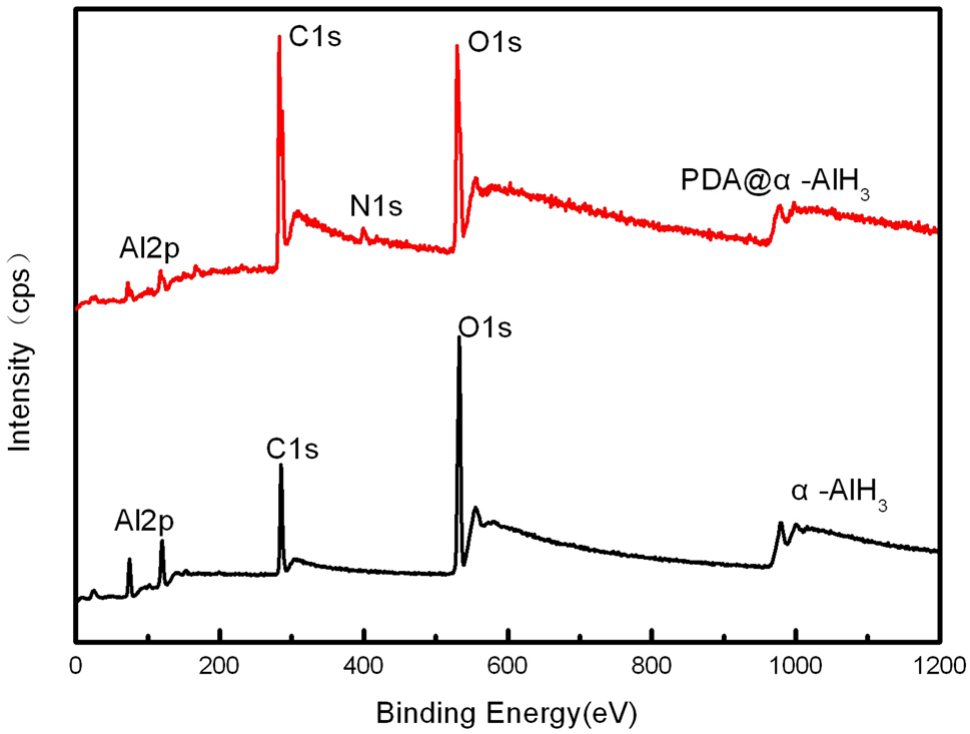
XPS patterns of α-AlH_3_ and PDA@α-AlH_3_.

The surface compositions of PDA@α-AlH_3_ and α-AlH_3_ were analyzed by photoelectron spectroscopy (XPS), and the results are shown in Fig. 2 and Table 1. Figure 2 shows that the element types on the surface of α-AlH_3_ before and after coating changed. The surface elements of α-AlH_3_ before coating only contain three elements: C, Al, and O. After coating, the surface elements of PDA@α-AlH_3_ contained not only three elements: C, Al, and O but also the characteristic N element peak of the polydopamine film. These observations indicate that the surface of α-AlH_3_ was successfully coated with a polydopamine film. However, the intensity of the peaks has changed. The intensity of the C1 s peak is significantly increased, indicating that the intensity of the O1 s peak and Al2p corresponding to the carbon element in the PDA coated on the surface of α-AlH_3_ is significantly reduced. At the same time, it can be seen more specifically from Table 1 that the content of C1 s increased from 18.17% to 52.37%, and the corresponding contents of O and Al were reduced. From Table 1, the content of O1 s is reduced from 38.58% to 22.83%, the content of Al2p is reduced from 43.25% to 21.27%, and the content of N1 s is characteristic of a polydopamine film at 3.53%, indicating that the surface of α-AlH_3_ is coated with PDA.

**Table 1:** XPS testing results of XPS of α-AlH_3_ and PDA@α-AlH_3_.

Sample	Al2p/%	C1 s/%	O1 s/%	N1 s/%
α-AlH_3_	43.25	18.17	38.58	0
PDA@α-AlH_3_	21.27	52.37	22.83	3.53

**Figure 3:**
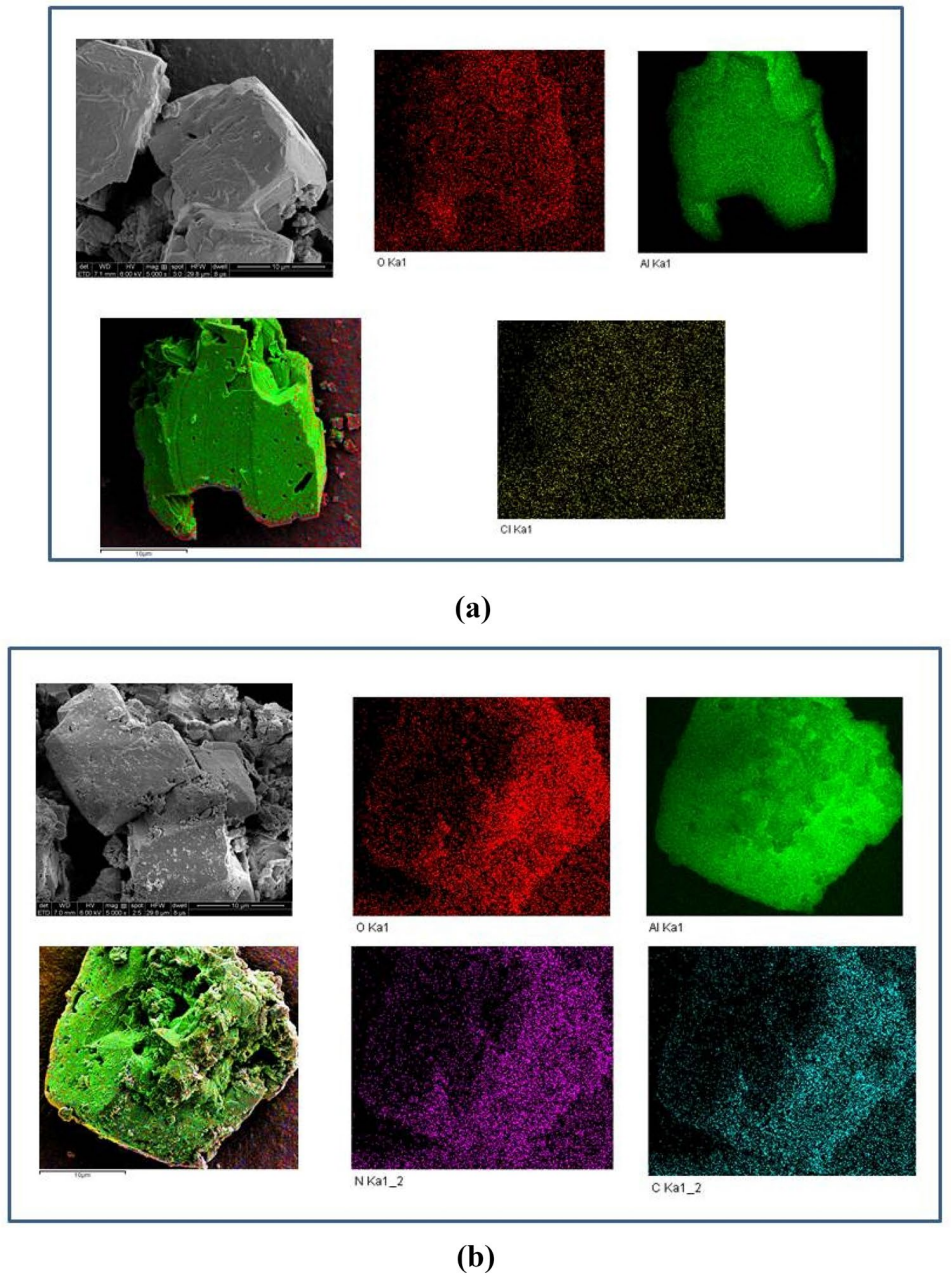
SEM and EDS mapping images of α-AlH_3_ and PDA@α-AlH_3_ collected from different location distributions, (**a**) α-AlH_3_, (**b**) PDA@α-AlH_3_.

To further study the influence of PDA on the micromorphology of α-AlH_3_, the morphology analysis results of PDA@α-AlH_3_ and α-AlH_3_ by SEM are shown in Fig. 3. Figure 3 shows the SEM images and atomic distribution as determined by the EDS mapping images of two samples obtained from different locations. Figure 3a is the SEM and EDS-mapping image of α-AlH_3_. It has a cubic morphology of polycrystalline irregularity, with a particle size of approximately 10 μm, a relatively smooth surface, and relatively sharp edges and corners. There is a superpositional phenomenon between the particles. Figure 3b is the SEM and EDS mapping image of PDA@α-AlH_3_. It is a cube with less regularity, with a particle size of approximately 10 μm. The surface is relatively uneven and is attached to polydopamine particles, indicating that PDA is evenly coated on the α-AlH_3_ surface. It is clear that the elements of α-AlH_3_ determined by EDS mapping are different in terms of their composition and distribution. EDS mapping shows that the Al shown in green is full of coverage Beause Al is the major element present in α-AlH_3_. The O distribution is shown in red patches and dots. The Cl distribution is shown in yellow patches and dots. This shows that some Cl minerals included in α-AlH_3_ have been unremoved by cleaning.

After coating, the elements of PDA@α-AlH_3_ contained not only Al and O but also the characteristic N and C elements of polydopamine. The C distribution is shown in blue patches and dots. The N distribution is shown in purple patches and dots. The composition of samples determined by EDS is given in Table 1. As seen from Table 2, the C atomic percentage was increased by 24.55%, and when the N atomic percentage was increased by 2.44%, α-AlH_3_ was coated by PDA, showing a significant enrichment of the C and N contents. At the same time, the Al and the O atomic percentages were decreased by 7.88% and 8.87%, respectively.

**Table 2:** Elemental analysis of α-AlH_3_ and PDA@α-AlH_3_ samples by EDS, n.d., not detected.

Elements	Al	O	Cl	N	C
Sample α-AlH_3_ mass %	87.22	12.49	0.29	n.d	n.d
Sample α-AlH_3_ atomic %	80.39	19.41	0.21	n.d	n.d
Sample PDA@α-AlH_3_ mass %	77.20	7.73	n.d	1.56	13.51
Sample PDA@α-AlH_3_ atomic %	62.47	10.54	n.d	2.44	24.55

Some studies have shown that dopamine first undergoes oxidation to dopaminequinone under alkaline conditions, followed by intramolecular cyclization via 1,4 Michael-type addition to yield leu-codopaminechrome. Leucodopaminechrome further undergoes oxidization and rearrangement to form 5,6-dihydroxyindole, which is easily oxidized to 5,6-indolequinone^28,29^. These two reaction products are capable of undergoing branching reactions, leading to the formation of multiple isomers of dimers and higher oligomers, which self-assemble through the reverse dismutation reaction between catechol and o-quinone to yield the cross-linked polymer^30^. A number of supramolecular interactions, including π-stacking, chargetransfer, and hydrogen bonding, have been shown to be the prominent features of the polymer’s structures^30^. In addition, with the increasing time, the coverage of PDA on the α-AlH_3_ surface increases due to conjugation of PDA aggregates on the surface, accompanying by the growth of PDA aggregates, which result in the deposition of PDA particles onto α-AlH_3_ surface. Therefore, compact and uniform PDA coating on α-AlH_3_ was possibly due to these factors. Firstly, under ambient conditions, polydopamine was prone to diffusion on organic α-AlH_3_ surfaces through noncovalent binding interaction such as hydrogen bonding, or π-π stacking to yield an effective adhesion layer; secondly, reactions between catechol in dopamine molecule and oxidation product o-quinone yielded the cross-linked polymers; thirdly, the cross-linked polymers can further assemble to PDA aggregates and deposit on the α-AlH_3_ surfaces, resulting in the PDA layers.

**Figure 4:**
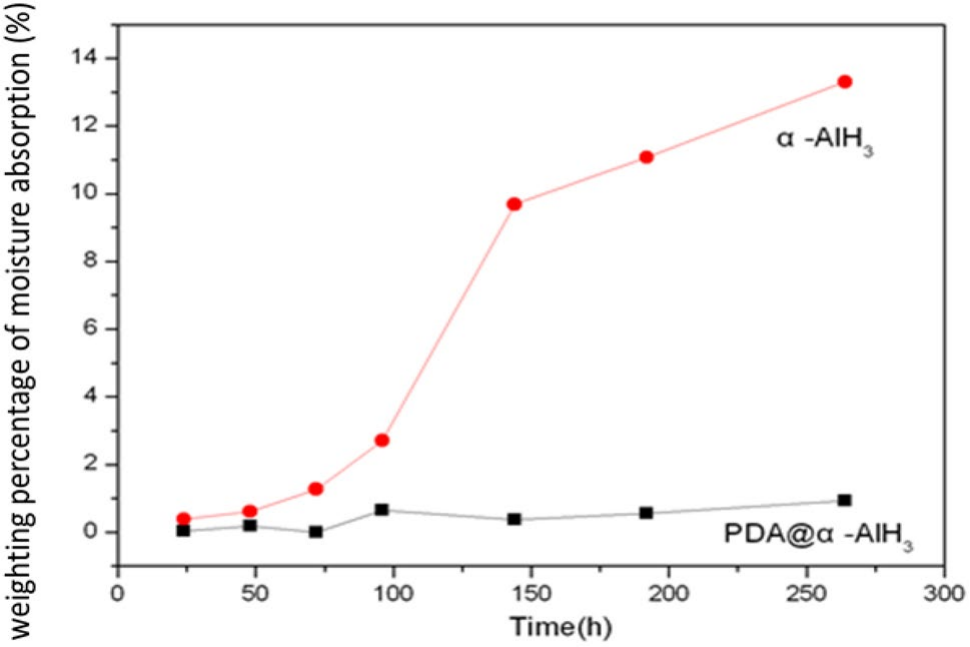
The moisture absorption curves of α-AlH_3_ and PDA@α-AlH_3_.

To study whether a small amount of coating agent PDA can slow down the moisture absorption of α-AlH_3_, the moisture absorption rates of PDA@α-AlH_3_ and α-AlH_3_ were tested. The water contact angles of PDA@α-AlH_3_ and α-AlH_3_ are measured. These results are shown in Fig. 4. The results show that at room temperature and 93% relative humidity, the moisture absorption rate of α-AlH_3_ coated with PDA is significantly lower than that of α-AlH_3_. With increasing time, the moisture absorption rate of uncoated α-AlH_3_ increases rapidly with storage time and reaches the equilibrium point of moisture absorption after 12 days, which is as high as 13.3%. The moisture absorption rate of α-AlH_3_ after being coated with PDA is only 0.5%, which shows that the polydopamine film on the surface plays an important role in isolating moisture in the air.

**Figure 5:**
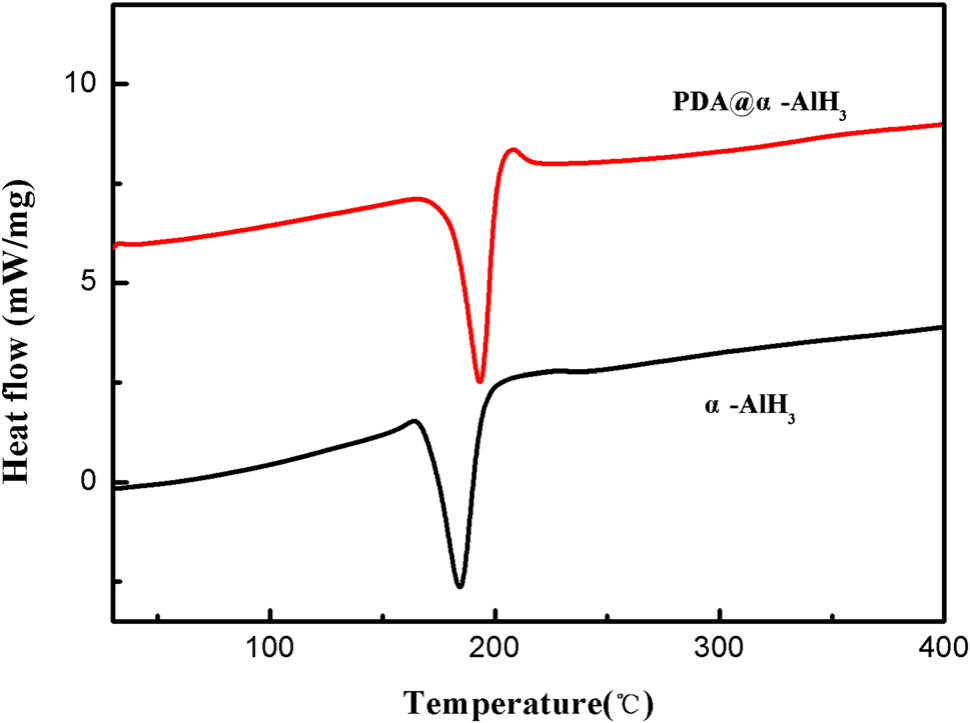
The DSC curves of α-AlH_3_ and PDA@α-AlH_3_.

Thermal stability is a key performance factor for novel materials. To investigate the thermal stability of the samples before and after modification, the thermal performance was determined by DSC. The test results of the samples are displayed in Fig. 5. The characteristic parameters are summarized in Table 3. As shown in Fig. 5, the DSC curve of α-AlH_3_ exhibits a single endothermic desorption process starting at and peaking at 181.2 °C, which is in accordance with the endothermic reaction being attributed to the dehydriding of α-AlH_3_. However, the thermal decomposition temperature of PDA@α-AlH_3_ is 191.1 °C, indicating that the thermal stability of α-AlH_3_ is greatly improved after PDA modification, which is due to the formation of organic PDA to improve its heat resistance. These results clearly demonstrate that PDA@α-AlH_3_ completely decomposed into Al is more difficult than pure α-AlH_3_.

**Table 3:** Thermal analysis data of α-AlH_3_ and PDA@α-AlH_3_.

Sample	Endothermic peak			
	T_o_/°C	T_P_/°C	T_e_/°C	*Δ*H/Jg^−1^
α-AlH_3_	166.2	181.2	204.1	405.1
PDA/α-AlH_3_	177.1	191.1	210.2	418.6

T_o_: onset temperature; T_p_: peak temperature of thermal profile; T_e_: end temperature; ΔH: total energy for endothermic profile.

According to the relevant literature, AlH_3_ will slowly decompose to hydrogen at room temperature. For this reason, the prepared samples are naturally placed in a desiccator, and after a period of time, the hydrogen content is tested, and the decomposition rate is indirectly calculated through the change in hydrogen content. The test results of samples α-AlH_3_ and PDA@α-AlH_3_ are shown in Table 4. Table 4 shows that the hydrogen content of the product is reduced due to the decomposition of. The initial hydrogen content of sample #1 is 9.745%. From the initial hydrogen content data of #2, #3 and #4, we can see that there are varying degrees of reduction in hydrogen about α-AlH_3_ modified by PDA. However, from the perspective of decomposition rate, the decomposition rate of α-AlH_3_ is the highest, and the decomposition rate is reduced after modification by PDA. The decomposition of PDA@α-AlH_3_ may be slowed due to the formation of the organic polymer PDA. The more organic content there was, the lower the decomposition rate.

**Table 4:** The H element content of α-AlH_3_ and PDA@α-AlH_3_ stored at room temperature.

No.	Sample	Storage time/d	Percent hydrogen/%		Decomposition rate/%
			Initial	windup	
#1	α-AlH_3_	365	9.745	9.632	1.16
#2	PDA/α-AlH_3_	365	9.136	9.126	0.11
#3	PDA/α-AlH_3_	365	9.095	9.089	0.07
#4	PDA/α-AlH_3_	365	9.235	9.215	0.22

To investigate the influence of PDA on the sensitivity of α-AlH_3_, different batches of prepared PDA@α-AlH_3_ samples were numbered, and the impact, friction and electrostatic sensitivity were tested to study the influence of PDA on the sensitivity of α-AlH_3_. The impact sensitivity was conducted according to the GJB772A-97 standard method 601.1. The friction sensitivity was conducted according to the GJB772A-97 standard method 602.1. The electrostatic sensitivity was evaluted according to the GJB5891.27-2006.^18^ The test results are shown in the Table 5.

**Table 5:** The sensitivity results of α-AlH_3_ and PDA@ AlH_3_.

No	sample	Impact sensitivity (%)	Friction sensitivity	Electrostatic sensitivity
#1	α-AlH_3_	14	40% (90°3.92 MPa)	V_50_ = 10.0 kV no fire
#2	PDA/α-AlH_3_	12	44% (90°3.92 MPa)	V_50_ = 10.0 kV no fire
#3	PDA/α-AlH_3_	14	44% (90°3.92 MPa)	V_50_ = 10.0 kV no fire
#4	PDA/α-AlH_3_	14	40% (90°3.92 MPa)	V_50_ = 10.0 kV no fire
#5	PDA/α-AlH_3_	12	40% (90°3.92 MPa)	V_50_ = 10.0 kV no fire

Experimental batch number #1 is α-AlH_3_, and experimental batch numbers #2, #3, #4 and #5 are all PDA-modified materials. From Table 5, it can be seen that the α-AlH_3_ surface modification material polydopamine has an effect on α-AlH_3_. The impact sensitivity, friction sensitivity and electrostatic sensitivity have little effect. The reason is that PDA did not change the crystal morphology, structure and crystal surface energy of α-AlH_3_. After being modified by PDA, it can meet the requirements of later application indexes.

## Conclusion

Polydopamine (PDA) was successfully generated on the surface of α-AlH_3_ by in situ dopamine (DA) polymerization, and the structure and morphology of the package were characterized by various test methods, such as XRD, XPS and SEM. The results showed that the coating layer was more uniform, the coating effect was better, and the crystal type of the material was not changed. The stability of the PDA@α-AlH_3_ composite at room temperature and 93% high humidity is significantly higher than that of uncoated α-AlH_3_. This research provides new ideas for the long-term storage performance of α-AlH_3_ and its application in propellants.

## Methods and materials

α-AlH_3_ was typically synthesized following a wet chemical method. LiAlH_4_ and AlCl_3_ react in diethyl ether to produce an alane-ether complex. Then, α-AlH_3_ was crystallized from the crystallization solution by removing ether by heating the crystallization solution to a temperature ranging from approximately 80 ℃ to 90 °C; additionally, the crystallization additive was also added to the crystallization solution. α-AlH_3_ was synthesized in our institute. Dopamine hydrochloride was purchased from Sigma–Aldrich and used as received. The other chemicals were commercial, analytical grade and used without further purification.

### Coating of α-AlH_3_ with PDA

**Figure 6:**
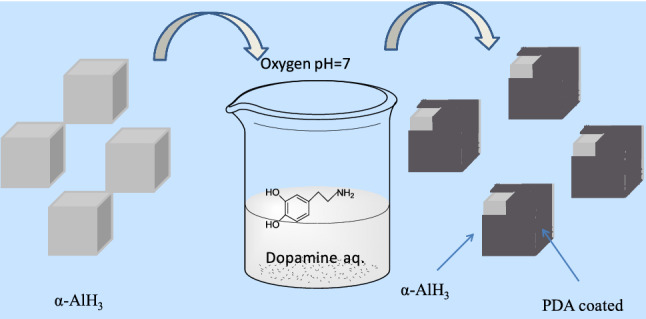
Possible deposition process of PDA@α-AlH_3_.

A phosphate-buffered saline (PBS pH = 7 ~ 7.5) solution was first prepared after the as-prepared solution and α-AlH_3_ was dispersed to the PBS solution, with stirring at 300 rpm for 30 min. Then, dopamine was added to the above suspension, with stirring at 200 rpm for 4 h. During the polymerization process, the color of the solution changed from white to dark brown as a result of dopamine polymerization. The obtained dark brown solution was filtered. The samples were rinsed with distilled water and then dried in a vacuum oven at 60 °C. The entire operation process is shown in Fig. 6.

### Hygroscopicity test

Static hygroscopicity refers to the GJB772A-97 method. Place the saturated solution of potassium nitrate in a desiccator. After equilibrium, a hygrometer was used to determine the relative humidity in the desiccator to be 93%. Combine α-AlH_3_ and PDA@α-AlH_3_ at the same time. Place it in a dry place, and use a Mettler analytical electronic analytical balance for weighing, with an accuracy of 0.0001. The weight change was recorded every 24 h, the change in moisture absorption rate was observed, and the moisture absorption rate was calculated as follows:

$$Q = \frac{{\nabla {\text{G}}}}{{\text{m}}} \times 100\%$$

In the formula, *Q* is the moisture absorption rate, ΔG is the weight gain of the sample after moisture absorption, and m is the initial weight of the sample.

### Characterization

Structural characterization of the *α-AlH*_**3**_ and PDA@*α-AlH*_**3**_ samples was performed by powder X-ray diffraction (XRD, DMAX2400 with *Cu K*_*a*_ radiation at *λ* = 1.5418 Å). The morphology of the samples was examined by field emission scanning electron microscopy (SEM, Quanta600FEG). The surface chemistry was analyzed using X-ray elemental analysis (XPS, Thermo Fisher spectrometer equipped with monochromatic Al Ka radiation (1486.6 eV)). The content of H element was analysised using organic element analyzer. The thermal analysis was studied by DSC (NETZSCH STA 449C). The samples were heated from room temperature to the set temperatures with a heating rate of 10 ℃/min under an Ar_2_ gas flow rate of 70 ml/min to prevent oxidation.
